# Effect of Green Synthesized Fe_3_O_4_NP Priming on Alfalfa Seed Germination Under Drought Stress

**DOI:** 10.3390/plants14081236

**Published:** 2025-04-18

**Authors:** Xinyue Wang, Mengting Ge, Xueqing He

**Affiliations:** College of Grassland Agriculture, Northwest A&F University, Yangling 712100, China

**Keywords:** alfalfa, drought stress, green synthesis, seed priming, seed germination

## Abstract

Drought stress is one of the key environmental factors restricting the germination of alfalfa seeds (*Medicago sativa* L.). Nanopriming is an innovative seed-priming technology able to meet economic, agronomic, and environmental needs in agriculture. However, the use of conventional nanomaterials is hampered by high costs, environmental risks, and biotoxicity. In this study, we synthesized iron oxide nanoparticles (Fe_3_O_4_NPs) using seasonal *Ginkgo biloba* leaf extracts (collected from August to November) obtained via an enzymatic ultrasonic-assisted method. The synthesized Fe_3_O_4_NPs were characterized using SEM, EDS, DLS, FTIR, UV-Vis, and XRD. To investigate the effects of Fe_3_O_4_NP priming on alfalfa seed germination under drought stress, germination and pot experiments were conducted with five Fe_3_O_4_NP priming concentrations (unprimed, 0, 20, 40, and 60 mg/L) and three PEG-6000 concentrations (0%, 10%, and 15%) to simulate normal, moderate, and severe drought conditions. The results showed that leaf extracts collected in November exhibited the highest flavonoid content (12.8 mg/g), successfully yielding bioactive-capped spherical Fe_3_O_4_NPs with a particle size of 369.5 ± 100.6 nm. Germination experiments revealed that under severe drought stress (15% PEG-6000), the 40 mg/L Fe_3_O_4_NP treatment most effectively enhanced seed vigor, increasing the germination rate, vigor index, and α-amylase activity by 22.1%, 189.4%, and 35.5% (*p* < 0.05), respectively, compared to controls. Under moderate drought stress (10% PEG-6000), the 20 mg/L Fe_3_O_4_NP treatment optimally improved germination traits, increasing the germination rate by 25.5% and seedling elongation by 115.6%. The pot experiments demonstrated morphological adaptations in alfalfa seedlings: under moderate drought stress, the 40 mg/L Fe_3_O_4_NPs significantly increased lateral root numbers, while under severe drought stress, the 60 mg/L Fe_3_O_4_NPs increased the root surface area by 20.5% and preserved the roots’ structural integrity compared to controls. These findings highlight that Fe_3_O_4_NPs synthesized via Ginkgo leaf extracts and enzymatic ultrasonic methods exhibit promising agricultural potential. The optimal Fe_3_O_4_NP priming concentrations enhanced seed vigor, germination traits, and drought resistance by modulating root morphology, with concentration-specific efficacy under varying drought intensities.

## 1. Introduction

Global climate change has exacerbated the reduction in water content in soil, leading to drought stress in plants, which seriously affects crop yields and quality [1]. This phenomenon has been shown to induce a state of drought stress in plants, thereby compromising their growth and development [2,3]. Alfalfa, a widely planted crop around the world due to its high protein content and palatability [4], is mostly distributed in arid and semi-arid regions in the north of China [5]. As a result, moisture has become a major factor affecting its germination. Since seed germination represents a pivotal stage in the life history of flowering plants [6], finding effective ways to improve germination under drought stress is of great significance. One such emerging approach is nanopriming. Nanopriming, as an emerging seed pretreatment method, is receiving increasing attention for improving the germination potential of seeds under drought stress [7]. It has been shown that the germination rate, seedling growth and vigor, seed water uptake, and the amylase activity of wheat seed germination and aging maize seed germination under drought stress were significantly increased by nanopriming [8].

As nanoparticles play a crucial role in nanopriming, the synthesis method of these nanoparticles becomes important. The green synthesis method is used to prepare metal nanoparticles by extracting the reducing substances from plant extracts through ultrasound and enzymatic digestion to reduce the metal ions in metal salt solutions [9]. Compared with industrial chemical synthesis, the green synthesis of nanomaterials has been gaining more and more attention in scientific research and industrial applications due to its environmental friendliness, biocompatibility, simplicity of preparation, and high-efficiency performance [10]. The green synthesis method utilizing plant extracts has gradually gained widespread attention. Ravikumar et al. [11] used the active substances from pomegranate (*Punica granatum* L.) peel and successfully prepared NiFe-NPs. Deng et al. [12] synthesized iron nanoparticles by green synthesis from sunflower (*Helianthus annuus* L.). Wang et al. successfully synthesized Ag-NMs and Fe_2_O_3_ NMs by using tea leaf residue and pod extract. Flavonoids in *G. biloba* have reducing properties and can be used as a natural reducing agent. Dong successfully prepared Ag-NPs/GBE, Pd-NPs/GBE, and AuNPs/GBE through *G. biloba*.

Although recent studies have focused on the green synthesis and characterization of nanoparticles, there remains a significant knowledge gap regarding their application in nanopriming for drought-stressed alfalfa. To address this limitation, we hypothesize that iron nanoparticles (Fe_3_O_4_NPs) synthesized using *G. biloba* flavonoids (collected in November) can enhance alfalfa seed resilience to drought stress through improved germination efficiency and amylase activation. Here, we present a threefold objective: (1) to develop an eco-friendly synthesis protocol utilizing the ultrasonic-assisted enzymatic extraction of *G. biloba* flavonoids across different phenological stages; (2) to optimize Fe_3_O_4_NPs’ fabrication parameters using the one-factor method; and (3) to systematically evaluate the priming effects of these biogenic nanoparticles on alfalfa germination metrics (the germination rate and vigor index) and drought-responsive biochemical markers (α-amylase activity and root traits) under drought conditions. This study aims to establish a novel green nano-technology approach for improving the drought tolerance of alfalfa.

## 2. Results

### 2.1. Extraction of Flavonoids in G. biloba

**Figure 1:**
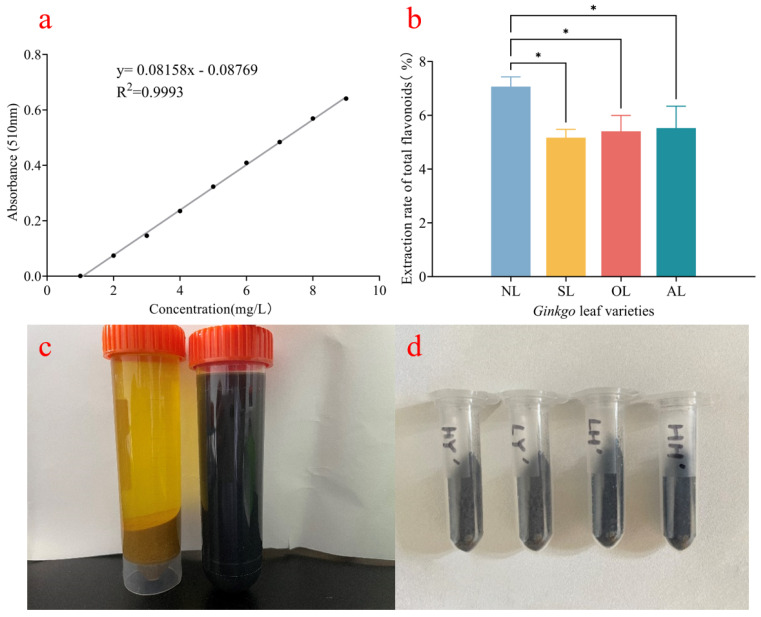
Green synthesized Fe_3_O_4_NPs. (**a**) Rutin standard curve; (**b**) The data in the figure are presented as mean ± standard deviation. * indicates significant differences between treatments (*p* < 0.05). *G. biloba* leaf types are denoted as follows: NLs for fallen yellow leaves, SLs for unshed yellow leaves, OLs for mixed yellow leaves, and ALs for green leaves. (**c**) The right side is the ginkgo extract solution, and the left side is the green synthesized nanoscale iron preparation solution; (**d**) Nanoscale iron extracted from different ginkgo leaves using ultrasonic-assisted enzymatic extraction method.

Fe_3_O_4_NPs were successfully synthesized using *G. biloba* leaf extract by the ultrasonic-assisted enzymatic extraction method. As shown in Figure 1a, under the concentration range of 0–10 mg/mL of Rutin standard solution, the flavonoids and absorbance had a good linear relationship, which conformed to the following linear fitting equation: y = 0.0816x − 0.0877 and the correlation coefficient R^2^ = 0.9993. Therefore, the concentration is in this range, and the application of this linear regression equation is meaningful. During the synthesis process, the color of the mixture containing a 1:20 ratio of the extract and FeSO_4_·7H_2_O changed from yellow to black after overnight shaking at 37 °C as shown in Figure 1c. The color change indicated that the phytoconstituents of *G. biloba* leaf extract caused the reduction of Fe into Fe_3_O_4_NPs. In Figure 1b, different periods of *G. biloba* had a significant effect on the content of total flavonoids in the extract. Under the ultrasonic-assisted enzymatic method, the NL group extracted the highest mean value of the absorbance of the extract, which was significantly different from all other groups, with an extraction rate of 7.1%, while there was no significant difference between SLs, OLs, and ALs, which were reduced by 26.9%, 23.5%, and 21.8%, respectively, compared with the NL group. Therefore, by comparing the flavonoid yields, it is clear that the use of NLs as a raw material for the extraction of the active substances from *G. biloba* is more efficient under the ultrasonic-assisted enzymatic extraction method. The total flavonoids in the *G. biloba* leaves at different periods were extracted by the ultrasound-assisted enzymatic method, and the extracts are shown in Figure 1c. A 2 mM FeSO_4_ solution was added drop by drop to the extract, and the green synthesized Fe_3_O_4_NPs were obtained by vertexing and oscillating them for 1 min, then leaving them to dry, as shown in Figure 1d.

### 2.2. Results of Nanomaterial Characterization

By calculating the yield of the total flavonoids extracted from the *G. biloba* leaves at four different periods, the NLs with the highest yield were selected for nanomaterial characterization.

#### 2.2.1. SEM, EDS, and DLS Characterization

**Figure 2:**
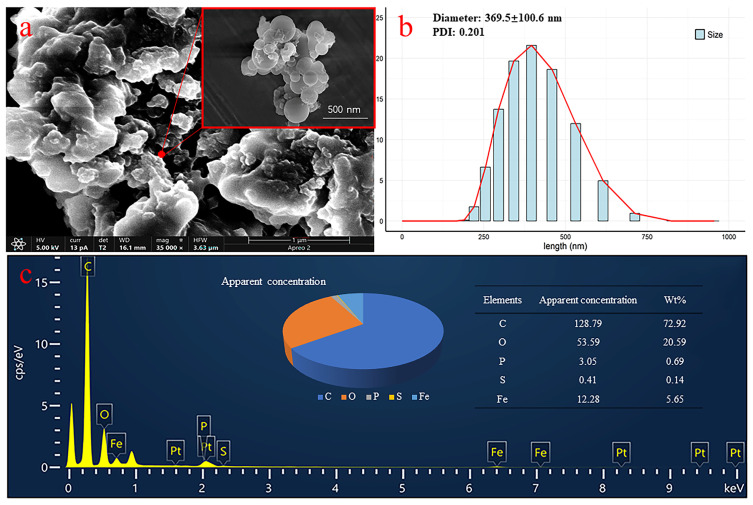
SEM, EDS, and DLS results of green synthesized Fe_3_O_4_NPs (NLs). (**a**) SEM image of the synthesized Fe_3_O_4_NPs at 35 KX and 50 KX magnification; (**b**) DLS size distribution of green synthesized Fe_3_O_4_NPs; (**c**) EDS spectrum and apparent concentration of elements of green synthesized Fe_3_O_4_NPs.

Scanning electron microscopy (SEM) was used to observe the microscopic morphology of Fe_3_O_4_NPs (NLs) with differing periods of *G. biloba* leaf extraction, and the results are shown in Figure 2a. It can be seen that the green synthesized Fe_3_O_4_NPs samples have a poorly dispersed microscopic morphology of spherical nanoparticles, with a more homogeneous distribution of particle size. Measured after the ethanol dispersion, the size distribution of the Fe_3_O_4_NPs obtained by the DLS analysis showed that the particles had an average diameter of 369.5 ± 100.6 nm with a polydispersity index (PDI) of 0.201 (Figure 2b). Qualitative and quantitative information about the elements on the sample’s surface or near-surface were obtained through energy-dispersive X-ray spectroscopy. Quantitative information and the results are shown in Figure 2c. Since green synthesis technology is based on plant tissues, it contains a higher content of protein constituent elements of C, N, O, P, and S. It can be seen through the energy spectra that the green synthesized nanomaterials also maintain a high level of Fe_3_O_4_NPs.

#### 2.2.2. FTIR Characterization

**Figure 3:**
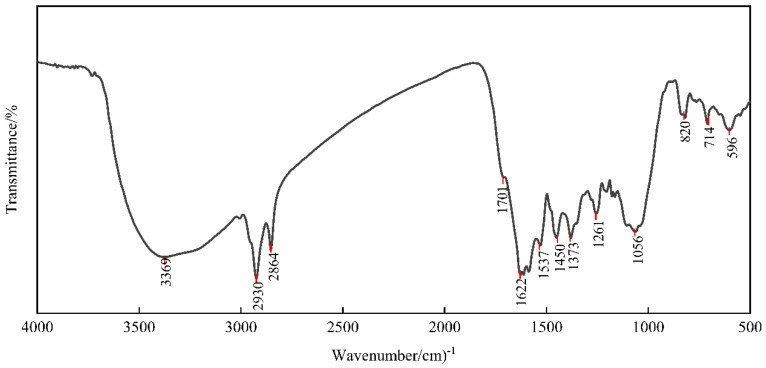
FTIR results of green synthesized Fe_3_O_4_NPs (NLs).

An FTIR analysis was used in order to determine the vibration characteristics of chemical functional groups in the green synthesized Fe_3_O_4_NPs. The FTIR spectral results listed in Table 1 include the wavenumber, absorbance, absorption band strength, and band assignments. The FTIR of the resultant material showed an absorption pattern similar to amino acid ligands in the 500–4000 cm^−1^ region. Moreover, there are surface functional groups in the interaction of metals because the *G. biloba* leaves are rich in polyphenols, acid derivatives, and proteins [13]. Figure 3 indicates the FTIR spectra of the *G. biloba* leaf extract of Fe_3_O_4_NPs. The curves showed that there was a major peak at 3369 cm^−1^ corresponding to the O-H stretching vibrations, which mainly act as reducing agents for the NPs’ synthesis. The peak at 1701 cm^−1^ in Figure 3 revealed the involvement of C=O stretching vibrations of acid derivatives, which act as a capping agent. The peaks at 1622 cm^−1^ and 1537 cm^−1^ are generally C=C stretching vibrations, possibly from an aromatic structure. Those at 1261 cm^−1^ and 1056 cm^−1^ are C-O stretching vibrations. The characteristic band of Fe-O is at 596 cm^−1^, indicating Fe_3_O_4_.

#### 2.2.3. UV-Vis-NIR and XRD Characterization

**Figure 4:**
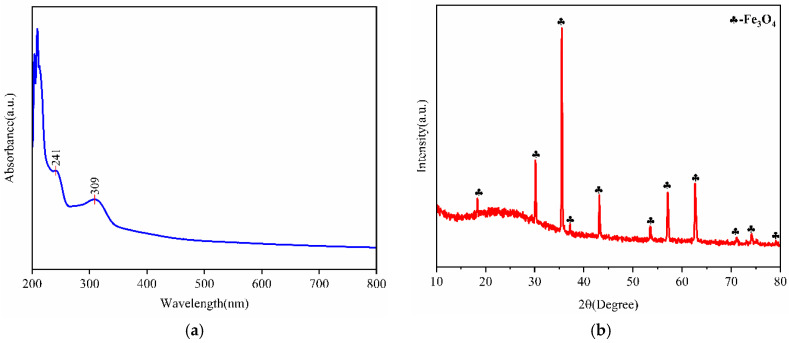
UV-Vis-NIR and XRD characterizations of green synthesized Fe_3_O_4_NPs (NLs). (**a**) UV-Vis-NIR results of green synthesized Fe_3_O_4_NPs; (**b**) XRD results of green synthesized Fe_3_O_4_NPs.

In addition to their chemical compositions and morphological and structural properties, for the catalytic application of the materials, their optical properties are significant, primarily absorption in the UV-Vis range of the spectrum [14]. As shown in Figure 4a, the UV-Vis absorption spectra of the green synthesized Fe_3_O_4_NPs based on *G. biloba* leaves were observed in the range of 200–800 nm. The absorption spectroscopy of the synthesized dried sample showed an absorption band at 241 and 309 nm, indicating the presence of both Fe_3_O_4_NPs and phytochemicals in *G. biloba* leaves. The absorption at 241 nm originates from the coordination interactions between Fe^3+^ ions and bioactive constituents (e.g., polyphenols) in the *G. biloba* extract [15]. The 309 nm peak unequivocally corresponds to Fe_3_O_4_NPs, aligning with the characteristic band-edge absorption of magnetite (300 nm) documented in the literature [16]. Combined with the morphology analysis and FTIR result, the synthesized nanoparticles were determined to be Fe_3_O_4_NPs.

The synthesized Fe_3_O_4_NPs exhibited high crystallinity with well-defined diffraction peaks characteristic of the face-centered cubic phase (JCPDS 04-009-4225). As shown in Figure 4b, prominent peaks corresponding to the (111), (220), (311), (222), (400), (422), (511), (440), and (533) crystallographic planes were observed at 2θ = 18.3°, 30.1°, 35.4°, 37.1°, 43.1°, 53.4°, 57.0°, 62.5°, and 74.0°, respectively. This well-defined crystalline structure results from the controlled alkaline synthesis conditions (37 °C and a pH of 9.0), in which the reductive phytoconstituents in the plant extract effectively mediated nanoparticle formation, consistent with previous reports [17,18]. The literature suggests that flavonoid compounds in plant extracts serve dual roles as reducing agents for Fe^2+^ ions and stabilizers preventing particle aggregation, ultimately facilitating crystalline-phase development [19]. Notably, the observed diffraction patterns align with the crystallographic features reported in green synthesized Fe_3_O_4_NPs systems, particularly those mediated by *Solanum lycopersicum* leaf extracts [15], confirming the cubic magnetite structure formation under optimized biosynthetic conditions.

### 2.3. Germination Parameters

#### 2.3.1. Two-Factor ANOVA

In order to further investigate the influence of drought stress and priming concentration on the seed germination of alfalfa, a two-factor ANOVA was performed, which included the SS, df, mean squares (MS), F-values, and *p*-values for both the drought stress and priming treatments, offering a comprehensive evaluation of their effects on seed germination. The analysis results are presented in Table 2, which indicates that the priming concentration had a significant influence on the germination characteristics of the developing seeds (*p* < 0.01). The germination characteristics of the alfalfa seeds remained relatively constant during the drought stress concentrations, varying from 0% to 15%, suggesting that the priming concentration may significantly affect the seed germination rate of alfalfa.

#### 2.3.2. Effect of Green Synthesized Fe_3_O_4_NPs on Alfalfa Seed Germination Under Drought Stress

**Figure 5:**
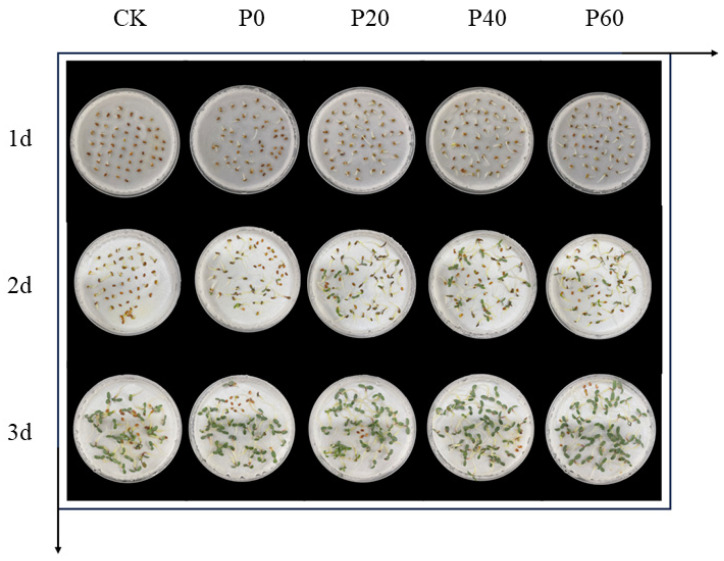
Effects of green synthesized Fe_3_O_4_NPs on the germination of alfalfa seeds under drought stress.

Figure 5 shows the morphological changes of the alfalfa seeds over the first three days of their germination after the priming of the green synthesized Fe_3_O_4_NPs. As can be seen in Figure 5, the morphological changes of the seeds on the first day of germination showed that the seeds with the priming treatment absorbed water for a short period of time and rapidly progressed through the stage of seed radicle emergence to form primary buds. Most of the embryonic axis was elongated to about 1 cm, while most of the seeds in the control group were in the absorbing period and did not break through the seed coat. Only a few of the seeds had ruptured seed coats. On the second day of seed germination, it could be clearly seen that the seeds of the triggered P0, P20, P40, and P60 treatment groups grew faster, the radicle elongated to 2–3 cm, and the cotyledons unfolded, whereas some of the seeds in the control group had not yet germinated. On the third day of seed germination, the cotyledons of the seeds in all treatment groups had already unfolded, and at this time, it could be seen that the treatment group with the initiated green synthesized Fe_3_O_4_NPs had a higher germination rate than the control group. The priming of the green synthesized Fe_3_O_4_NPs could promote the seed germination rate to a great extent. The alfalfa seeds after the priming treatment sprouted faster and the embryonic root elongation was faster. Therefore, the priming of the green synthesized Fe_3_O_4_NPs had a greater promotional effect on the seed germination speed and embryonic root length.

#### 2.3.3. Effects of Green Synthesized Fe_3_O_4_NPs on Alfalfa Seed Germination Parameters Under Drought Stress

Table 3 shows the effect of the green synthesized Fe_3_O_4_NP priming treatment on the germination parameters of the alfalfa seeds under drought stress. The results showed that the green synthesized Fe_3_O_4_NP priming had a significant effect on the germination potential, germination rate, germination index, and vigor index of the alfalfa seeds (*p* < 0.05). The promotion of alfalfa seed germination by the Fe_3_O_4_NP priming treatments without added stress treatments reached a maximum at P20. Compared with CK, the germination rate significantly (*p* < 0.05) increased by 19.2%, the germination potential significantly (*p* < 0.05) increased by 25.5%, the germination index significantly (*p* < 0.05) increased by 115.6%, and the vigor index significantly (*p* < 0.05) increased by 742.3% at P20. Notably, the germination index significantly (*p* < 0.05) increased by 119.5% at P40. However, the increase in germination percentage and germination potential of the P20, P40, and P60 seeds was not significant. For the drought stress treatment of 15% PEG-6000, there were significant (*p* < 0.05) differences between the treatments with different priming concentrations, and a more obvious trend of “increasing and then decreasing” was observed, with the seed germination rate, germination percentage, germination index, and vigor index of P40 reaching a peak that was significantly higher than the rest of the treatment groups. In contrast, a significant (*p* < 0.05) decrease in the germination potential, germination rate, and germination index was observed in P0 compared to the control group (CK). Therefore, the green synthesized Fe_3_O_4_NPs may have played an important role in alfalfa seed germination.

### 2.4. Effect of Green Synthesized Fe_3_O_4_NP Priming on Alfalfa Seed α-Amylase Activity

**Figure 6:**
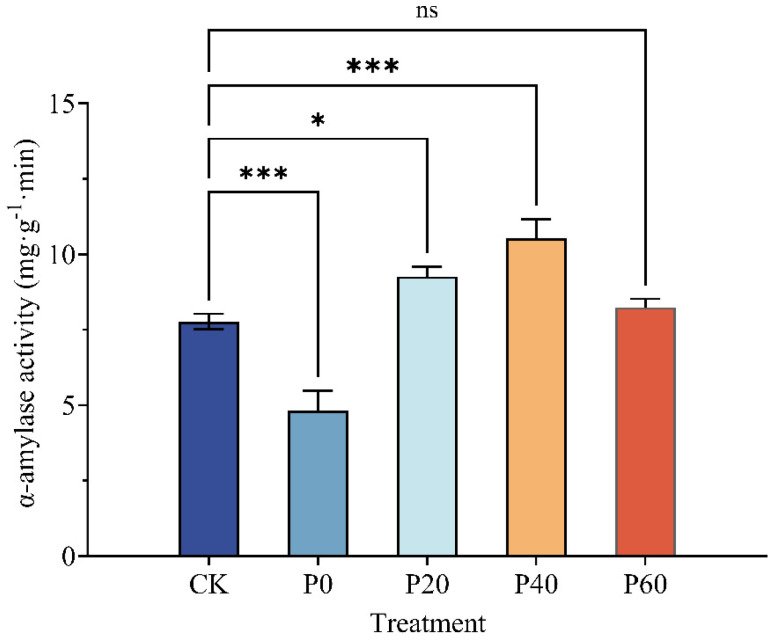
Effects of green synthesized Fe_3_O_4_NPs on the α-amylase activity of alfalfa seeds (* 0.01 ≤ *p* < 0.05, *** *p* < 0.001, ns means no significant difference).

Figure 6 shows that the alfalfa amylase activity varied with treatment concentration during the alfalfa seed germination. The α-amylase activity of the water-primed alfalfa seeds was significantly (*p* < 0.05) reduced by 37.9% compared with the control, and P60 did not significantly promote α-amylase activity. In particular, the α-amylase activity of P40 was significantly (*p* < 0.05) increased by 35.5% over the control. Therefore, the green synthesized Fe_3_O_4_NP priming may have increased the α-amylase activity of the alfalfa seeds, and the priming treatment concentration of 40 mg/L was the most significant, followed by 20 mg/L.

### 2.5. Effect of Green Synthesized Fe_3_O_4_NP Priming on Root Surface Area of Alfalfa

**Figure 7:**
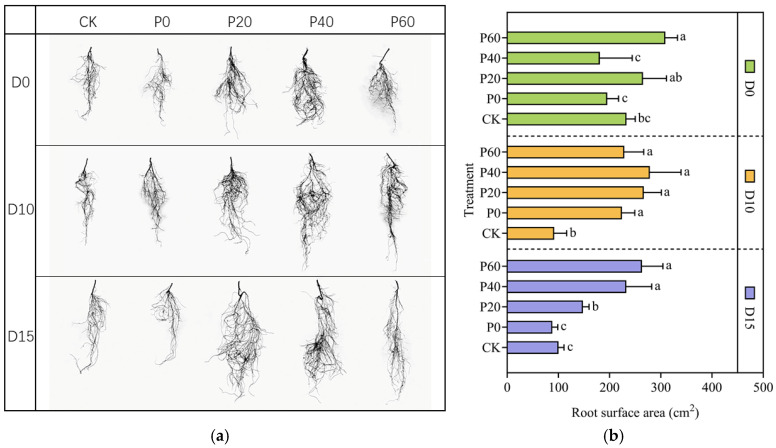
Effects of green synthesized Fe_3_O_4_NPs on roots of alfalfa under drought stress. (**a**) Root morphology of different treatments; (**b**) Root surface area of different treatments. Different lowercase letters represented significant differences between different treatments under drought stress (*p* < 0.05).

**Figure 8:**
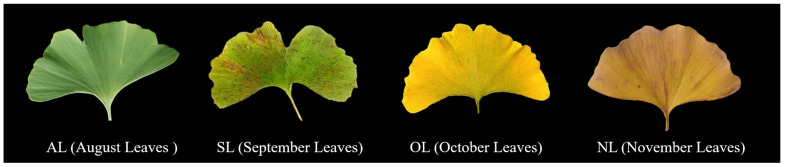
Four different periods of *G. biloba* leaves.

Figure 7 shows scanning images of the alfalfa seedlings’ roots and the effects of the green synthesized Fe_3_O_4_NPs on the root surface area of the alfalfa under drought stress. Table 4 indicates the effects of the green synthesized Fe_3_O_4_NPs on the root average diameter of the alfalfa under drought stress. The root phenotypic responses to the green synthesized Fe_3_O_4_NP priming varied significantly across the PEG-6000 gradients (Figure 8). Under 0% PEG-6000, P60 increased the total root surface area by 45.6%, from 195.37 cm^2^ in the control group to 309.04 cm^2^ (*p* < 0.01), with an average diameter of 0.80 mm. The treatment with 10% PEG-6000 enhanced the efficacy of Fe_3_O_4_NPs: P40 had a maximum surface area of 278.51 cm^2^, which was 203% higher than the control group (*p* < 0.001), while the diameter decreased by 11.6%, indicating that the proliferation of fine roots was enhanced, which is beneficial for water absorption. However, under 15% PEG-6000 conditions, P60 showed double optimization, with an increase of 20.5% in surface area compared to the drought stress control group, which reached 263.32 cm^2^, and 15.9% in diameter, which increased to 0.80 mm.

## 3. Discussion

*G. biloba* has received much attention in agriculture because it contains nearly 100 flavonoids with high antioxidant activity [20]. A study on the antioxidant properties of *G. biloba* extracts found that the DPPH and ABTS free radical clearance rates were high, and the extracts also showed a significant reducing ability [21] and could be used as reducing agents in green synthesized nanomaterial assays. Recent research studies demonstrate that conventional methods for extracting flavonoids, including methanol, ethanol, and water extraction, are intricate and time-consuming, yielding low returns [22]. In contrast, the ultrasound-assisted enzymatic method has emerged as a productive technique to enhance the yield of flavonoids. Ultrasonic-assisted extraction was used to extract the total flavonoids from *Z. bungeanum* residue [23]. This method augments the mechanical and cavitation effects of ultrasound, leading to the destruction of biological structures such as cell walls and cell membranes and promoting intracellular mass transfer. The enzymes further accelerate the destruction of cell walls and cell membranes, facilitating flavonoid release and extraction. In this study, an experimental investigation was conducted on the flavonoid extraction rate of *G. biloba* leaves at different time periods while maintaining a fixed extraction temperature of 40 °C, an extraction period of 20 min, an ethanol concentration of 68%, and an extraction power of 218 W. The period of the *G. biloba* leaves had an effect on the total flavonoid extraction rate, which tended to decrease at first and then increase with the passage of time. The highest total flavonoid extraction rate of 7.1% was achieved with *G. biloba* leaves that had naturally fallen off and were collected in November. A similar result was previously reported in Sangut and Samec [24]. Consequently, NLs (leaves collected in November) were selected for the preparation of the *G. biloba* extract.

Nanoparticles have unique biological properties due to their large specific surface area [25]. Compared to traditional physicochemical synthesis methods, the green synthesis of nanoparticles using plant extracts from natural and non-toxic plants has developed as a new trend [26]. For instance, studies have demonstrated the successful synthesis of nanoparticles using extracts from various plants, such as ZnO NPs synthesized from chia seeds [27], AuNPs synthesized from *Halodule uninervis* [28], and FeNPs synthesized from *Eucalyptus globulus* leaf [29] and Oolong tea, green tea, and black tea [30]. In this study, Fe_3_O_4_NPs were synthesized by using *G. biloba* extract from NLs as a reducing agent. The color of the extract before and after the reaction changed from light yellow to dark brown, which shows the successful synthesis of Fe_3_O_4_ NPs, as depicted in Figure 1c. This initial reduction creates a nucleation center, which leads to the accumulation of more metal ions while also incorporating the nucleation site next to it [31]. Nanoparticles are formed as a result, which become entrapped with biological molecules of the plant for better stability and an improved morphology. We further proved the successful synthesis of Fe_3_O_4_NPs (with a size of 369.5 ± 100.6 nm) by nanomaterial characterization. Comprehensive characterization techniques, including SEM, EDS, DLS, FTIR, XRD, and UV-Vis, were employed to ascertain the optical properties, crystalline structure, and morphology of the synthesized Fe_3_O_4_NPs.

The enhanced germination index under drought stress ensures synchrony between early root development and residual soil moisture availability, which is vital for activating stress-resilient traits and sustaining biomass accumulation in perennial alfalfa systems [32,33]. Plants emerging from seeds with higher germination indices exhibit a greater capacity to survive recurrent drought events. In this study, the green synthesized Fe_3_O_4_NPs significantly enhanced alfalfa seed germination metrics—including the germination rate, germination potential, vigor index, and α-amylase activity—under drought stress conditions. Under non-drought conditions, the green synthesized Fe_3_O_4_NPs enhanced the germination index by 19.2% compared to untreated seeds (*p* < 0.05). This improvement likely stems from the gradual release of bioavailable iron ions, which promote early enzymatic activation and energy metabolism during germination. However, no significant phytotoxicity was observed, suggesting the biocompatibility of green synthesized Fe_3_O_4_NPs in standard agricultural settings. These findings align with research by Siddiqui et al. [34], who reported that selenium nanoparticles improved barley seed germination by modulating physiological and biochemical pathways. Similarly, Alkhatib et al. [35] observed that priming tobacco seeds with 20 nm Fe_3_O_4_ nanoparticles (at a 10 mg/L concentration) activated antioxidant systems and osmotic regulators, thereby enhancing germination efficiency. This consistency across studies underscores the broad applicability of nanoparticle-mediated seed priming for mitigating abiotic stress responses.

This study revealed the drought-intensity-dependent regulation of alfalfa root morphology by green synthesized Fe_3_O_4_NP priming. Under well-watered conditions, 60 mg/L Fe_3_O_4_NPs significantly increased the total root surface area (*p* < 0.01) by enhancing root biomass accumulation while maintaining a stable root diameter, aligning with the findings of Ndou et al. [36] in *Sorghum bicolor*, for which Fe_3_O_4_NPs improved its water uptake capacity. Under moderate drought stress, priming with 40 mg/L Fe_3_O_4_NPs enhanced fine-root proliferation and reduced the root diameter, consistent with the classic strategy of optimizing root architecture to enhance water-foraging efficiency [37]. However, under severe drought stress, priming with 60 mg/L Fe_3_O_4_NPs had a unique adaptive response: the roots prioritized structural reinforcement through an increased diameter rather than surface area expansion. This contrasts sharply with the generalized “drought-induced root thinning” pattern proposed by Dong et al. [38], suggesting that Fe_3_O_4_NPs may remodel root developmental patterns via mechanisms such as carbon reallocation or cell wall modification. Green synthesized Fe_3_O_4_NPs, stabilized by plant extracts rich in flavonoids, play critical roles in plant physiological processes, particularly osmotic regulation and root cell division. Flavonoids, as plant secondary metabolites, are key mediators of biotic and abiotic stress responses. Recent studies have shown that flavonoid accumulation enhances plant drought resistance: under osmotic stress, plant roots produce osmoregulatory substances to maintain cell turgor [39].

While this study demonstrates that green synthesized Fe_3_O_4_NPs improve drought tolerance in *Medicago sativa* L. by enhancing seed germination and seedling root growth, the underlying molecular mechanisms (e.g., osmotic regulation or root cell division) remain incompletely characterized. Future research could systematically compare the effects of green synthesized and chemically synthesized nanoparticles on alfalfa physiology and gene expression under drought conditions to clarify whether the superior drought mitigation observed with green nanoparticles stems from their surface bioactive compounds (e.g., flavonoids) or structural properties. Such studies would deepen our understanding of green-nanoparticle-mediated stress tolerance and guide the rational design of bio-inspired nanomaterials for sustainable agriculture, particularly for enhancing crop resilience to climate-induced water scarcity. While the current work focuses on germination enhancement, future investigations will employ surface-sensitive characterization to quantify the extract’s role in nanoparticle stabilization and biological activity regulation.

## 4. Materials and Methods

### 4.1. Materials

The experiment was conducted in 2023 at the College of Grassland Agriculture, Northwest A&F University. *G. biloba* leaves were collected from the South Campus of Northwest Agriculture and Forestry University, Yangling Demonstration Zone, Xianyang City, Shaanxi Province. Alfalfa seeds (Queen) were purchased from Jiangsu Thousand Flowers and Hundred Charms Seed Industry Co.

### 4.2. Preparation of the Green Synthesized Fe_3_O_4_ NPs

#### 4.2.1. Preparation of *G. biloba* Extract

*G. biloba* leaves (Figure 8) at four different periods were collected for analysis: ALs (August leaves), SLs (September leaves), OLs (October leaves), and NLs (November leaves). The *G. biloba* leaves were thoroughly rinsed with distilled water to remove the fine dust particles and dried in an oven at 60 °C for 2 h, then ground into a powder using a grinder and filtered through a 60-mesh sieve to obtain homogeneous sample powder. An amount of 1 g of *G. biloba* powder was mixed with 20 mL of 68% ethanol and 0.8 g of composite enzyme (cellulase:pectinase = 1:1). Then, the mixture was ultrasonicated at 40 °C and 218 W for 20 min, and the supernatant was taken as *G. biloba* extract [40].

#### 4.2.2. Determination of Total Flavonoids in *G. biloba* Extracts

First, 20.00 mg of Rutin standard was accurately weighed, and the solution was fixed with 30% ethanol solution in a 100 mL volumetric flask and shaken well to obtain 0.2 mg/Rutin standard solution. Accurately measure 0.0, 1.25, 2.5, 3.75, 5, 6.25, 8.75, and 10 mL of Rutin standard solution in 25 mL volumetric flask. Add 0.75 mL of 30% NaNO_2_ solution, shake it well, and let it stand for 6 min. Add 0.75 mL of 10% Al(NO_3_)_3_ solution, shake it well, and let it stand for 6 min. Add 10 mL of 4% NaOH solution and 30% ethanol solution to obtain 25 mL at the scale line, shake it well, and let it stand for 15 min. The absorbance was measured at 510 nm. The concentration of Rutin standard solution was taken as the horizontal coordinate, the absorbance value was taken as the vertical coordinate, and the standard curve of Rutin was plotted. Centrifuge the *G. biloba* extract and determine the absorbance of ginkgo extract referring to the above method. By combining this with the standard curve and Formula (1), the total flavonoid content in the extract can be derived [23,41].

(1) $$Total\;flavonoid\;content\left(mg/g\right)=\frac{V\times C\times N}{M}$$

where V is the total volume of extract (mL); C is the concentration of sample solution (mg/mL); N is the dilution factor; and M is the mass of *G. biloba* powder (g).

#### 4.2.3. Synthesis and Characterization of Nanomaterials

Fe_3_O_4_NPs are prepared through a low-cost and eco-friendly method [42]. In the reaction procedure, 2 mM FeSO_4_ solution was combined with the prepared *G. biloba* extract, followed by vortex oscillation for 1 min. It was left to stand to ensure complete homogenization, after which the pH was adjusted to 9 using NaOH, and the mixture was oscillated overnight at 37 °C. The attained black product is washed with 68% ethanol, dried in an oven at 60 °C for 12 h, and kept in a stoppered bottle for further use. The nanoparticles were stored in a desiccator protected from light. Surface morphology and particle size were observed by scanning electron microscopy (SEM, Thermo Fisher Scientific Apreo 2S+, Waltham, MA, USA) and dynamic light scattering (DLS, Malvern Zetasizer ZS 90, Malvern, Worcestershire, UK). Energy-dispersive X-ray spectroscopy (EDS, OXFORD Ultim Max 65, Oxford, UK) was used to analyze qualitatively and quantitatively the types and contents of elements in the micro-regions. To analyze the surface functional group characteristics, FTIR measurements of green synthesized nanoparticles and prepared sample were taken with Thermo Nicolet iS5 spectrometer, Waltham, MA, USA. X-ray diffraction (XRD, Malvern Panalytical Empyrean, Malvern, Worcestershire, UK) with Cu–Ka radiation using a wavelength of 1.52 Å and Jade 5.0 software were used to detect and match diffraction peaks and observe crystal-phase structures. Ultraviolet–visible spectroscopy (UV-Vis, Shimadzu 3600-plus, Kyoto, Japan) was used to determine optical absorption characteristics.

### 4.3. Seed Priming Experiment

#### 4.3.1. Nanopriming

To ensure a uniform and efficient green synthesis of nanoparticles, we selected *G. biloba* NLs with the highest flavonoid extraction rate as the seed priming material. Alfalfa seeds with uniform size and full grains were treated with various concentrations of green synthesized Fe_3_O_4_NP priming solution (0, 20, 40, and 60 mg/L). Untreated alfalfa seeds were used as the control and labeled as control. Seeds were immersed in priming media for 24 h in the dark at 15 °C, and the proportion of seed weight to priming solution was 1:5 g/mL. Seeds were dried at room temperature after rinsing them 2–3 times with distilled water.

#### 4.3.2. Seed Germination Test

Seed germination test was carried out using the top-of-paper method and PEG-6000 (0%, 10%, and 15%) to simulate drought stress. The 50 alfalfa primed seeds were placed in Petri dishes (12 cm in diameter) lined with double-layered moist filter paper, and three biological replicates were performed in the experiments. The above were placed in an artificial insemination incubator at 20 ± 2 °C, a relative humidity of 85%, a photoperiod of 16/8 h (light/dark), and 10,000 Lx radiation for germination test [43]. Table 5 shows the codes and treatments in this research.

After germination test, the data of seeds and seedlings such as shoot length, root length, germination index, and vigor index were measured. Seed germination percentage, germination potential, germination index, vigor index, mean germination time [44], germination speed index [45], germination peak value [46], and final germination percentage [47] were calculated according to the following formula:

(2) $$\begin{array}{c} \mathrm{Germination}\;\mathrm{rate}\left(GR\right)=\frac{\mathrm{total}\;\mathrm{number}\;\mathrm{of}\;\mathrm{germinated}\;\mathrm{seeds}}{\mathrm{total}\;\mathrm{number}\;\mathrm{of}\;\mathrm{seeds}\;\mathrm{for}\;\mathrm{testing}}\times 100\% \end{array}$$

(3) $$\begin{array}{c} \mathrm{Germination}\;\mathrm{potential}\;(\mathrm{GP})=\frac{\mathrm{number}\;\mathrm{of}\;\mathrm{germinated}\;\mathrm{seeds}\;\mathrm{in}\;\mathrm{the}\;\mathrm{first}\;3d}{total\;number\;of\;seeds\;for\;testing}\times 100\% \end{array}$$

(4) $$\begin{array}{c} \mathrm{Germination}\;\mathrm{index}\;(\mathrm{GI})=\sum \frac{number\;of\;sprouted\;seeds\;on\;day\;i}{day\;i} \end{array}$$

(5) $$\begin{array}{c} \mathrm{Vigor}\;\mathrm{index}\;\left(VI\right)=\mathrm{germination}\;\mathrm{index}\left(GI\right)\times \mathrm{seedling}\;\mathrm{length} \end{array}$$

(6) $$\begin{array}{c} \mathrm{Mean}\;\mathrm{germination}\;\mathrm{time}\;(\mathrm{MGT})=\frac{\sum_{i=1}^{k}N_{i}T_{i}}{\sum_{i=1}^{k}N_{i}} \end{array}$$

(7) $$\begin{array}{c} \mathrm{Germination}\;\mathrm{peak}\;\mathrm{value}\;\left(GPV\right)=\max(\frac{N_{1}}{T_{1}},\frac{N_{2}}{T_{2}},\cdots ,\frac{N_{i}}{T_{i}}) \end{array}$$

(8) $$\begin{array}{c} \mathrm{Final}\;\mathrm{germination}\;\mathrm{percentage}\;\left(FGP\right)=\frac{Number\;of\;germinated\;seeds}{Total\;number\;of\;seeds} \end{array}$$

(9) $$\begin{array}{c} \mathrm{Germination}\;\mathrm{speed}\;\mathrm{index}\;\left(GSI\right)=\frac{\sum_{i=1}^{k}\frac{N_{i}}{T_{i}}}{100} \end{array}$$

where $N_{i}$ is the number of germinated seeds until the ith day $T_{i}$ from the first to the last day of germination (k) in days^−1^.

### 4.4. Determination of α-Amylase Activity

The 3,5-Dinitrosalicylic acid color development method was used to determine the amylase activity of sprouted seeds. Weigh 0.1 g of alfalfa seeds that have sprouted at 25 °C for two days and have a bud length of 1 cm, and then place them in a mortar, add 2 mL distilled water, grind them into a homogenate, divide them into 10 mL centrifugal tubes with distilled water with a fixed volume of 10 mL, immerse them for 15 min, and shake them every 2 min, so that they are fully extracted. Centrifuge them at 4000 r·min^−1^ at 4 °C for 10 min, and extract the supernatant as amylase stock solution. Aspirate 2 mL of amylase stock solution into a 10 mL centrifuge tube and then dilute it to 10 mL with distilled water to obtain the total amylase solution. The amylase stock solution was used to passivate β-amylase at a high temperature of 70 °C. After adding reagents according to the experimental method, a bath of boiling water was used for 10 min and then cooled rapidly. The absorbance value at 540 nm was measured using a UV spectrophotometer, and the corresponding activity was calculated according to the following formula:

(10) $$\begin{array}{c} \alpha -\mathrm{amylase}\;\mathrm{activity}=\frac{\left(A-A^{\prime }\right)\times V_{t}}{FW\times V_{s}\times t} \end{array}$$

where A: mg of maltose produced by α-amylase hydrolysis of starch; $A^{\prime }$: maltose content in the α-amylase control tube; Vs: volume of enzyme solution used in color development; Vt: total volume of the diluted solution of the sample; FW: sample weight (g); and t: enzyme action time (min).

### 4.5. Root Traits

All collected fine roots were thoroughly rinsed under running water in preparation for subsequent measurements of relevant indices. The cleaned roots were placed in transparent Petri dishes and scanned using a high-resolution digital scanner (Epson V600, 6400 × 9600 dpi, Nagano, Japan) to obtain high-resolution images. The obtained images were then uploaded into the root analysis system (WinRHIZO Pro 32-bit 2020a, Quebec City, QC, Canada) to extract data on root length, root surface area, root average diameter, and root volume.

### 4.6. Data Analysis

The data were analyzed by Microsoft Excel 2021 and SPSS 27 processing system. Multiple comparisons between data were performed, such as two-way analysis of variance (ANOVA) and LSD. Graphs were plotted using Origin 8.0 and tabulated using Microsoft Excel 2021.

## 5. Conclusions

This study demonstrated that the *G. biloba* leaf extracts collected in November exhibited the highest flavonoid content (12.8 mg/g) with an extraction rate of 7.1%, establishing them as the optimal raw material for flavonoid extraction. The characterization confirmed the successful synthesis of spherical iron nanoparticles (Fe_3_O_4_NPs) with a particle size of 50–80 nm, and the EDX analysis revealed the presence of carbon and oxygen elements on their surfaces. Under severe drought stress (15% PEG), the 40 mg/L Fe_3_O_4_NP treatment significantly enhanced alfalfa seed vigor (*p* < 0.05), showing a superior germination rate, vigor index, and α-amylase activity compared to controls. Under moderate drought stress (10% PEG), the 20 mg/L Fe_3_O_4_NP treatment exhibited the most pronounced improvement in germination traits. The pot experiments revealed that the 40 mg/L Fe_3_O_4_NPs significantly increased the lateral root density in seedlings under moderate drought stress, while the 60 mg/L Fe_3_O_4_NPs under severe drought stress increased the root surface area by 20.5% while maintaining root structural integrity. These findings highlight that Fe_3_O_4_NPs synthesized from *G. biloba* leaf extracts effectively improve alfalfa seed germination and drought resistance through nanopriming. Future research should focus on determining optimal concentration thresholds and elucidating the physiological and biochemical mechanisms underlying these enhancements.

## Figures and Tables

**Table 1:** FTIR results of green synthesized Fe_3_O_4_NPs (NLs).

Number	Wavenumber (cm^−1^)	Absorbance (Abs)	Absorption Band Strength	Band Assignments
1	596	0.121212	Weak	Fe-O Stretching
2	714	0.103149	Weak	C-H Stretching
3	820	0.096330	Weak	C-H Stretching
4	1056	0.402104	Medium	C-O Stretching
5	1261	0.334503	Medium	C-O Stretching
6	1373	0.412196	Medium	C-H Stretching
7	1450	0.430146	Medium	C-H Stretching
8	1537	0.453304	Medium	C=C or N-H Stretching
9	1622	0.614133	Strong	C=C Stretching
10	1701	0.232626	Medium	C=O Stretching
11	2846	0.440695	Medium	C-H Stretching
12	2930	0.617398	Strong	C-H Stretching
13	3369	0.498590	Medium	O-H Stretching

**Table 2:** Two-factor analysis of variance on the seed germination under different drought stress concentrations and priming concentrations.

Source	SS	df	Mean Square	F	P
Germination rate (GR)					
Drought stress concentration (D)	11,159.64	2	5579.82	389.89	3.39 × 10^−22^
Priming concentration (P)	5501.87	4	1375.47	96.11	1.17 × 10^−16^
D*P	2346.13	8	293.27	20.49	3.55 × 10^−10^
Germination potential (GP)					
Drought stress concentration (D)	8659.38	2	4329.69	369.01	7.52 × 10^−22^
Priming concentration (P)	7591.64	4	1897.91	161.75	7.65 × 10^−20^
D*P	2049.96	8	256.24	21.84	1.62 × 10^−10^
Germination index (GI)					
Drought stress concentration (D)	21,618.61	2	10809.31	745.43	2.66 × 10^−26^
Priming concentration (P)	11,065.53	4	2766.38	190.78	7.17 × 10^−21^
D*P	2614.37	8	326.8	22.54	1.09 × 10^−10^
Vigor index (VI)					
Drought stress concentration (D)	5330.48	2	2665.24	9.68	5.70 × 10^−4^
Priming concentration (P)	371,681.36	4	92920.34	337.5	1.79 × 10^−24^
D*P	80,256.32	8	10032.04	36.44	2.20 × 10^−13^
Mean germination time (MGT)					
Drought stress concentration (D)	6.29	2	1.57	32.63	1.56 × 10^−10^
Priming concentration (P)	2.79	4	1.40	28.95	9.94 × 10^−8^
D*P	2.99	8	0.37	7.75	1.36 × 10^−5^
Germination peak value (GPV)					
Drought stress concentration (D)	50.80	2	12.7	5.21	2.63 × 10^−3^
Priming concentration (P)	79.62	4	39.8	16.32	1.60 × 10^−5^
D*P	46.62	8	5.83	2.39	4.01 × 10^−2^
Final germination percentage (FGP)					
Drought stress concentration (D)	0.55	2	0.14	96.11	1.17 × 10^−16^
Priming concentration (P)	1.12	4	0.56	389.90	3.40 × 10^−22^
D*P	0.24	8	0.03	20.50	3.54 × 10^−10^
Germination speed index (GSI)					
Drought stress concentration (D)	0.15	2	0.04	183.48	1.26 × 10^−20^
Priming concentration (P)	0.36	4	0.18	892.68	1.87 × 10^−27^
D*P	0.04	8	0.01	25.34	2.51 × 10^−11^

Notes: df, degree of freedom; MS, mean square; F, F-ratio. *p* < 0.05 indicates a significant difference, *p* < 0.01 indicates an extremely remarkable difference, and the symbol ‘*’ represents the interaction effect between the two factors in the two-factor ANOVA.

**Table 3:** Effects of green synthesized Fe_3_O_4_NPs on the germination of a*lfalfa* seeds under drought stress.

Treatment	GR (%)	GP	VI	GI	MGT (d)	GPV	FGP	GSI
CK-D0	83.33 ± 8.33 b	60.00 ± 2.00 c	48.94 ± 3.48 c	210.02 ± 17.77 d	3.37 ± 0.16 a	5.95 ± 0.59 b	0.83 ± 0.08 b	0.17 ± 0.01 c
P0-D0	90.67 ± 1.15 ab	87.33 ± 6.43 b	98.69 ± 6.47 ab	250.26 ± 8.79 c	1.55 ± 0.30 b	10.98 ± 4.31 a	0.91 ± 0.01 ab	0.37 ± 0.02 b
P20-D0	99.33 ± 1.15 a	95.33 ± 1.15 a	105.51 ± 3.88 ab	365.91 ± 16.91 a	1.62 ± 0.14 b	8.43 ± 1.33 ab	0.99 ± 0.01 a	0.39 ± 0.02 ab
P40-D0	97.33 ± 3.06 a	90.67 ± 1.15 ab	107.44 ± 2.80 a	321.43 ± 16.06 b	1.60 ± 0.22 b	7.85 ± 1.35 ab	0.97 ± 0.03 a	0.41 ± 0.01 a
P60-D0	96.00 ± 5.29 a	88.00 ± 5.29 ab	96.38 ± 7.24 b	271.41 ± 18.63 c	1.91 ± 0.33 b	7.25 ± 0.98 ab	0.96 ± 0.05 a	0.36 ± 0.03 b
CK-D10	50.00 ± 0.00 c	46.00 ± 2.00 d	32.84 ± 0.63 d	151.63 ± 16.75 d	2.71 ± 0.06 a	4.17 ± 0.00 b	0.50 ± 0.00 c	0.10 ± 0.00 d
P0-D10	75.33 ± 1.15 b	70.00 ± 2.00 c	56.48 ± 1.94 c	265.04 ± 6.47 c	2.38 ± 0.15 b	6.81 ± 1.20 a	0.75 ± 0.01 b	0.19 ± 0.01 c
P20-D10	86.00 ± 2.00 a	84.00 ± 2.00 a	79.00 ± 2.83 a	362.05 ± 30.91 a	1.84 ± 0.06 c	7.66 ± 0.99 a	0.86 ± 0.02 a	0.27 ± 0.01 a
P40-D10	80.00 ± 4.00 ab	74.00 ± 5.29 bc	65.53 ± 5.37 b	370.09 ± 18.28 a	2.16 ± 0.21 b	6.83 ± 1.49 a	0.80 ± 0.04 ab	0.22 ± 0.02 b
P60-D10	86.67 ± 7.57 a	80.67 ± 6.11 ab	69.36 ± 3.30 b	313.85 ± 14.49 b	2.19 ± 0.13 b	7.42 ± 1.82 a	0.87 ± 0.08 a	0.23 ± 0.01 b
CK-D15	39.33 ± 1.15 d	33.33 ± 1.15 d	23.95 ± 1.02 c	45.71 ± 7.77 e	2.96 ± 0.23 a	3.12 ± 0.24 b	0.39 ± 0.01 d	0.08 ± 0.00 c
P0-D15	30.67 ± 1.15 e	27.33 ± 3.06 e	19.03 ± 1.83 d	153.58 ± 15.27 d	2.84 ± 0.47 ab	2.97 ± 0.68 b	0.31 ± 0.01 e	0.06 ± 0.00 c
P20-D15	68.00 ± 2.00 b	63.33 ± 1.15 b	50.17 ± 4.19 a	382.39 ± 13.65 b	2.38 ± 0.18 b	6.06 ± 0.82 a	0.68 ± 0.02 b	0.16 ± 0.02 a
P40-D15	78.00 ± 2.00 a	73.33 ± 2.31 a	54.63 ± 2.78 a	426.81 ± 14.31 a	2.50 ± 0.12 ab	6.33 ± 1.45 a	0.78 ± 0.02 a	0.17 ± 0.01 a
P60-D15	58.00 ± 4.00 c	55.33 ± 2.31 c	41.73 ± 2.58 b	323.28 ± 18.40 c	2.40 ± 0.17 b	5.70 ± 1.37 a	0.58 ± 0.04 c	0.13 ± 0.01 b

Notes: GR: germination rate; GP: germination potential; VI: vigor index; GI: germination index; MGT: mean germination time; GPV: germination peak value; FGP: final germination percentage; GSI: germination speed index. Values are means of three replicates ± standard deviation; means with different letters are statistically different (Duncan’s multiple comparison at *p* < 0.05).

**Table 4:** Effects of green synthesized Fe_3_O_4_NPs on root average diameter of alfalfa under drought stress.

	Control	P0	P20	P40	P60
D0	0.74 ± 0.03 a	0.71 ± 0.13 a	0.77 ± 0.11 a	0.68 ± 0.11 a	0.80 ± 0.12 a
D10	0.58 ± 0.07 b	0.68 ± 0.14 a	0.73 ± 0.08 a	0.68 ± 0.08 a	0.74 ± 0.09 a
D15	0.52 ± 0.07 a	0.53 ± 0.10 ab	0.67 ± 0.12 b	0.76 ± 0.10 c	0.80 ± 0.06 c

Notes: Values are means of three replicates ± standard deviation; means with different letters are statistically different (Duncan’s multiple comparison at *p* < 0.05).

**Table 5:** Seed priming test codes and treatments.

Drought Stress Concentration	Priming Concentration	Code
0% PEG-6000	Unprimed	CK-0
	0 mg/L	P0-0
	20 mg/L	P20-0
	40 mg/L	P40-0
	60 mg/L	P60-0
10% PEG-6000	Unprimed	CK-10
	0 mg/L	P0-10
	20 mg/L	P20-10
	40 mg/L	P40-10
	60 mg/L	P60-10
15% PEG-6000	Unprimed	CK-15
	0 mg/L	P0-15
	20 mg/L	P20-15
	40 mg/L	P40-15
	60 mg/L	P60-15

## Data Availability

Data are contained within the article.
